# Fibrin-Based Hydrogels with Reactive Amphiphilic Copolymers for Mechanical Adjustments Allow for Capillary Formation in 2D and 3D Environments

**DOI:** 10.3390/gels10030182

**Published:** 2024-03-06

**Authors:** Svenja Wein, Carina Schemmer, Miriam Aischa Al Enezy-Ulbrich, Shannon Anna Jung, Stephan Rütten, Mark Kühnel, Danny Jonigk, Wilhelm Jahnen-Dechent, Andrij Pich, Sabine Neuss

**Affiliations:** 1 Helmholtz Institute for Biomedical Engineering, BioInterface Group, RWTH Aachen University, Pauwelsstrasse 20, 52074 Aachen, Germany; wjahnen@ukaachen.de (W.J.-D.); sneuss-stein@ukaachen.de (S.N.); 2 Institute of Pathology, RWTH Aachen University, Pauwelsstrasse 30, 52074 Aachen, Germany; mkuehnel@ukaachen.de (M.K.); djonigk@ukaachen.de (D.J.); 3 Chair for Laser Technology LLT, RWTH Aachen University, Steinbachstraße 15, 52074 Aachen, Germany; c.schemmer@t-online.de; 4 Functional and Interactive Polymers, Institute of Technical and Macromolecular Chemistry, RWTH Aachen University, Worringerweg 1, 52074 Aachen, Germany; alenezy@dwi.rwth-aachen.de (M.A.A.E.-U.); sjung@dwi.rwth-aachen.de (S.A.J.); pich@dwi.rwth-aachen.de (A.P.); 5 DWI–Leibniz Institute for Interactive Materials, RWTH Aachen University, Forckenbeckstraße 50, 52074 Aachen, Germany; 6 Electron Microscopic Facility, University Clinics, RWTH Aachen University, Pauwelsstrasse 30, 52074 Aachen, Germany; sruetten@ukaachen.de

**Keywords:** fibrin-based hydrogels, cell-based angiogenesis, copolymer integration, in vitro pre-vascularization, biohybrid constructs

## Abstract

This study focuses on enhancing controllable fibrin-based hydrogels for tissue engineering, addressing existing weaknesses. By integrating a novel copolymer, we improved the foundation for cell-based angiogenesis with adaptable structural features. Tissue engineering often faces challenges like waste disposal and nutrient supply beyond the 200 µm diffusion limit. Angiogenesis breaks through this limitation, allowing the construction of larger constructs. Our innovative scaffold combination significantly boosts angiogenesis, resulting in longer branches and more capillary network junctions. The copolymer attached to fibrin fibers enables precise adjustment of hydrogel mechanical dynamic properties for specific applications. Our material proves effective for angiogenesis, even under suppression factors like suramin. In our study, we prepared fibrin-based hydrogels with and without the copolymer PVP12400-co-GMA10mol%. Using a co-culture system of human umbilical vein endothelial cells (HUVEC) and mesenchymal stem cells (MSC), we analyzed angiogenetic behavior on and within the modified hydrogels. Capillary-like structures were reproducibly formed on different surfaces, demonstrating the general feasibility of three-dimensional endothelial cell networks in fibrin-based hydrogels. This highlights the biomaterial’s suitability for in vitro pre-vascularization of biohybrid implants.

## 1. Introduction

**Figure 1:**
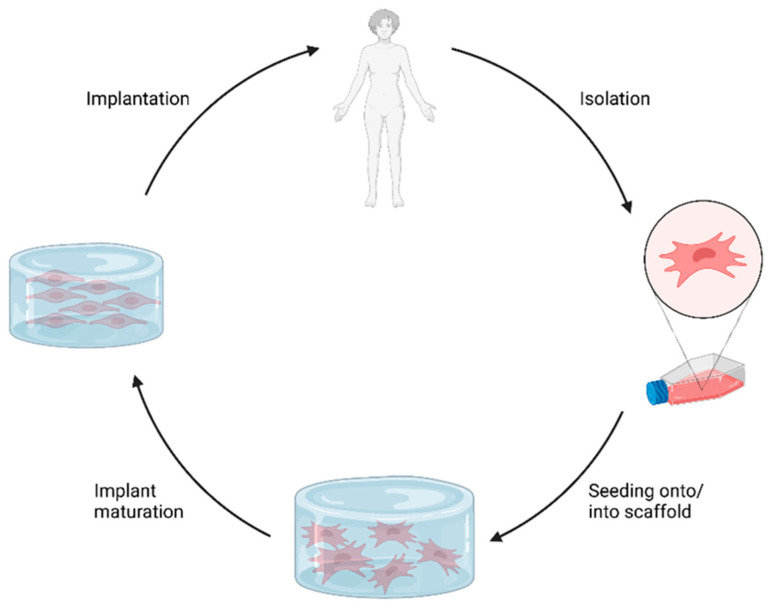
General concept of biohybrid tissue-engineered implants. Patient-derived cells (e.g., mesenchymal stem cells) are isolated and expanded in culture. Cells are seeded onto/into a scaffold material. Subsequent implant maturation comprises cell proliferation and differentiation in vitro. Finally, the matured construct is implanted. Created with biorender.com (accessed on 23 March 2023).

In tissue engineering, living cells combined with bioactive materials aim to create biohybrid constructs that can replace or regenerate damaged tissues when implanted. Recent methods focus on using patient-derived cells. The process involves isolating autologous cells, often stem cells, expanding them in culture, and seeding them onto scaffolds (Figure 1) [1]. These cells adapt to the new material, which can be influenced through growth factors or mechanical stimulation to encourage desired cell types [2,3].

However, tissue engineering’s clinical applications remain mostly limited to thin or avascular tissues, like cartilage [4]. One main challenge is the oxygen supply to cells within engineered constructs. Oxygen and nutrients rely on diffusion, meaning cells must be close to capillaries [5]. Promoting vascularization is crucial to ensure oxygen and nutrient delivery to cells within larger implants [4,6].

While spontaneous blood vessel growth from the host occurs after implantation, it is slow and can lead to insufficient oxygen supply [4], which might result in cell death or inconsistent responses. To overcome this, in vitro pre-vascularization is proposed. This involves creating capillary-like networks using endothelial cells on a fibroblast or mesenchymal stem cell feeder layer within a fibrin gel matrix [5,7].

Fibrin hydrogel offers several advantages as a scaffold material for tissue engineering. Firstly, it closely resembles the natural extracellular matrix, providing an environment conducive to cell attachment, proliferation, and differentiation. Additionally, fibrin hydrogels possess bioactive properties that promote angiogenesis and tissue regeneration, enhancing the healing process. Moreover, fibrin hydrogels are biocompatible and biodegradable, minimizing immune response and allowing for gradual replacement with newly formed tissue. However, fibrin hydrogels also have limitations, such as rapid degradation rates, which may affect their long-term stability and suitability for certain applications. Additionally, their mechanical properties may require enhancement for specific tissue engineering needs, necessitating the incorporation of reinforcing materials or modifications to improve mechanical strength.

Fibrin gel’s unique properties make it an excellent matrix for promoting angiogenesis (blood vessel formation) [8,9]. In both two-dimensional and three-dimensional settings, fibrin gel offers stability, porosity, and the possibility to incorporate growth factors [10]. In a three-dimensional environment, fibrin gel mimics the natural extracellular matrix, making it ideal for regenerative medicine [11,12].

Fibrin’s biodegradability allows gradual replacement of the scaffold with new tissue over time. However, its rapid degradation might limit long-term use. Polymer fibers can reinforce the scaffold, controlling its degradation profile for tissue regeneration [13]. Combining a fibrin-based hydrogel with 3D-printed polymer fibers offers a biocompatible, viscoelastic environment that promotes cellular attachment and mimics the natural extracellular matrix [10,14]. This approach accommodates cellular needs post-implantation.

This technique employs autologous sources for cells, fibrinogen, and thrombin, making it promising for generating fully autologous implants. This minimizes rejection and foreign body responses, potentially leading to clinical applications.

Fibrin hydrogels play a pivotal role in tissue engineering across medical fields. They expedite wound healing, aiding in chronic wounds, burns, and surgical incisions. In regenerative medicine, they deliver cells and growth factors, promoting tissue regrowth in bone, cartilage, skin, nerves, and vasculature.

Moreover, they act as carriers for controlled drug release, especially in localized therapies like cancer treatment. In surgery, fibrin hydrogels serve as adhesives, promoting tissue bonding and hemostasis, while improving implant integration.

Additionally, they are essential for creating organoid models and organ-on-a-chip platforms, mimicking native tissue environments for drug testing and disease modeling. With their adaptability, biocompatibility, and controllable properties, fibrin hydrogels are vital tools in advancing tissue engineering and regenerative medicine across diverse medical applications.

In summary, tissue engineering merges living cells with bioactive materials to create biohybrid constructs for tissue replacement. Patient-derived cells, especially stem cells, are used with scaffolds and growth factors to encourage desired cell types [15]. Vascularization is a challenge for larger constructs due to oxygen diffusion limitations. In vitro pre-vascularization using endothelial cells in a fibrin gel matrix addresses this issue. Fibrin’s properties promote angiogenesis and gradual scaffold replacement, although its rapid degradation is a concern. Polymer fibers reinforce the scaffold’s degradation profile and provide a biocompatible environment, with autologous sources making it a promising approach for clinical applications.

Scaffold materials are essential for wound healing, particularly in challenging cases. They provide structural support for tissue regeneration and guide cell behavior in a controlled manner, mimicking the natural extracellular matrix [16]. These materials often possess bioactive properties, releasing growth factors and cytokines to stimulate cell activity and tissue regeneration. Additionally, scaffold materials act as a protective barrier, shielding the wound from external threats and creating a conducive environment for healing. Among scaffold materials, hydrogels stand out due to their high water content, which promotes cell growth and facilitates nutrient exchange [17]. Their flexibility allows for tailored release of therapeutic agents, enhancing their utility in wound healing [18]. Scaffold materials, especially hydrogels, play a crucial role in promoting efficient wound healing by providing structural support, guiding cell behavior, and creating a protective environment for tissue regeneration.

**Figure 2:**
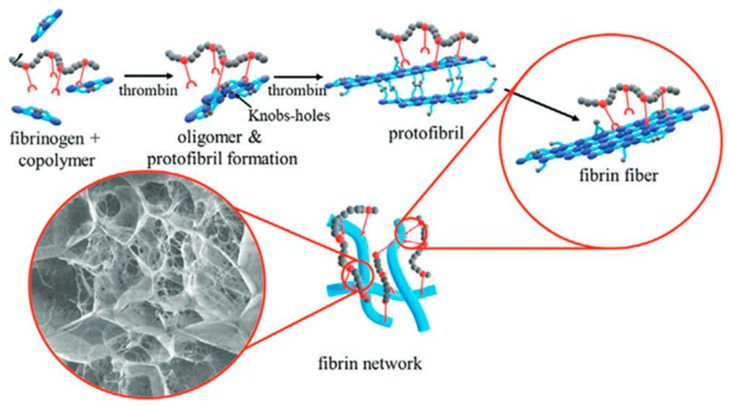
Figure and caption taken from the publication “Impact of Reactive Amphiphilic Copolymers on Mechanical Properties and Cell Responses of Fibrin-Based Hydrogels”, written by Al Enezy-Ulbrich et al. [15]. Formation of fibrin hydrogel from fibrinogen (blue rods) and a synthetic polymer (gray and red chain). The copolymers bind to the functional groups of the fibrinogen. Catalyzed by thrombin and via self-assembly, protofibrils are formed. The protofibrils assemble to fibrin-fibers.

To analyze this, we used new adjustable fibrin-based hydrogels, to which we linked the *N*-vinylpyrrolidone-based copolymer PVP_12400-co-_GMA_10mol%_, which was synthesized by RAFT polymerization [15,19]. The functional copolymer contains 10 mol% of glycidyl methacrylate (GMA) to enable covalent bond formation towards the amine and thiol groups of the fibrin (Figure 2).

### Impact Statement

The experiments presented here provide conclusive results regarding the influence of the mechanical controllability of fibrin hydrogels on capillarization. By using copolymers, we succeeded in obtaining a positive effect on capillary growth on and within hydrogels. This is an important starting point, for example, in the field of wound healing, as even larger poorly healing wounds can heal as quickly as possible and the tissues can be supplied with blood again. At the same time, this tissue engineering approach offers far-reaching possibilities about the growth of artificial organoids and organs to achieve a nutrient supply beyond the diffusion limit.

## 2. Results and Discussion

### 2.1. Rheology

**Figure 3:**
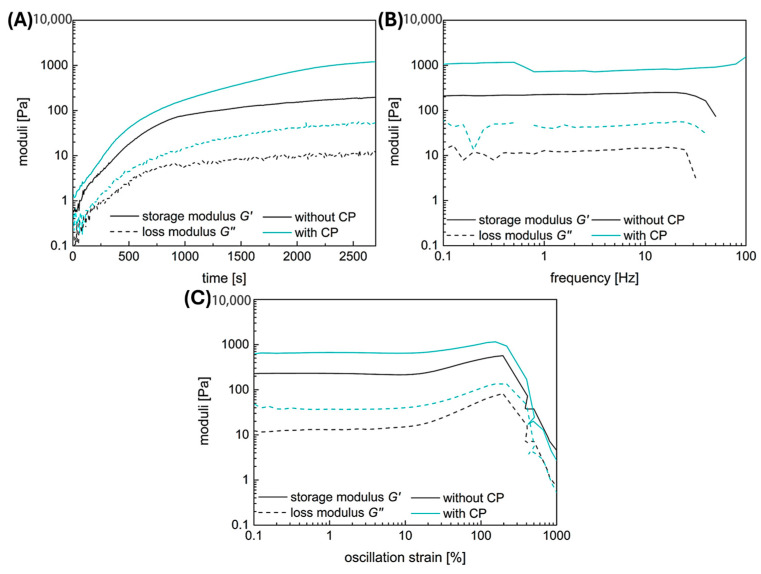
Characterization of the mechanical properties of fibrin-based hydrogels without and with functional copolymer (CP) by rheology: (**A**) time-dependent measurement, (**B**) frequency-dependent measurement, and (**C**) strain-dependent measurement.

Both hydrogel types, with and without the fibrin fiber copolymer, exhibit typical viscoelastic behavior akin to fibrin-based hydrogels, though with differing strengths. Results are shown in Figure 3, with gelation taking 45 min. Linear viscoelasticity ends around a critical strain of 10% for both samples. The functional copolymer enhances the storage modulus of fibrin-based hydrogels due to covalent crosslinks between the copolymer’s epoxy groups and fibrin’s thiols or amines [15]. Frequency-dependent measurements confirm the hydrogel nature of the enhanced system; storage and loss moduli remain independent of frequency until a critical value.

Both samples show slight strain-stiffening, though it is weaker in the copolymer-containing sample compared to pure fibrin. This is attributed to additional covalent bonds between the copolymer and fibrin, leading to a denser, less flexible network. While previous studies with lower copolymer concentrations showed enhanced strain-stiffening, higher copolymer concentration here leads to denser networks and increased storage modulus, yet limits fiber movement dynamics, resulting in weaker strain-stiffening behavior.

### 2.2. Capillary-like Structures on Hydrogels with and without Copolymer

The addition of the copolymer PVP_12400-co-_GMA_10mol%_ to the fibrinogen solution allows for the modulation of the gel’s mechanical properties, such as increased storage modulus, as well as its degradation rate, as described before by Al Enezy-Ulbrich et al. [15]. To investigate the behavior of HUVEC on these copolymer-modified fibrin gels, HUVEC were seeded on a layer of MSC on the hydrogel surface and the formation of capillary-like structures was observed.

**Figure 4:**
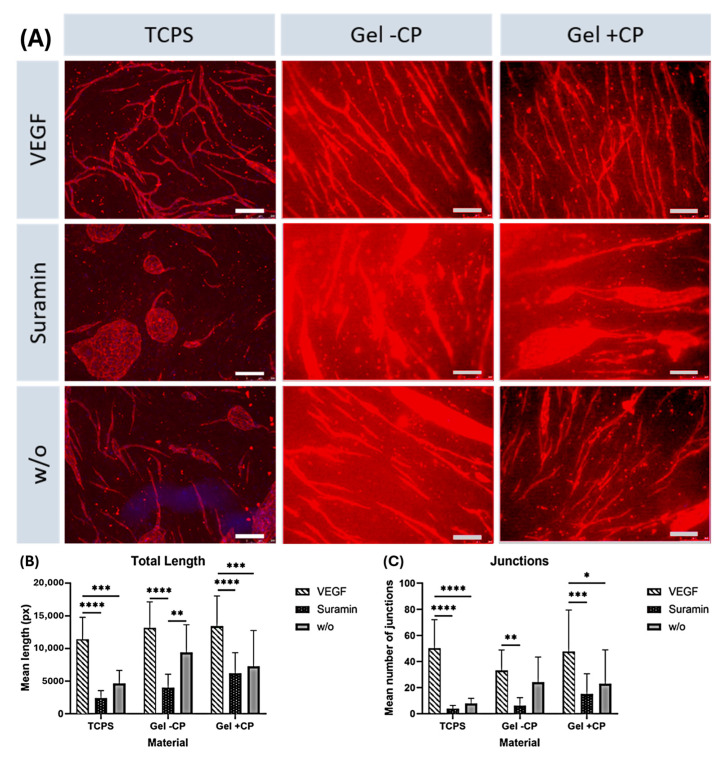
Capillary-like structures on TCPS and gels with and without copolymer (CP). (**A**) Immunofluorescence staining of CD31 (red); images were taken at 50× magnification. Scale bars: 250 µm. (**B**,**C**) Mean network length and number of junctions on each material per approach, as analyzed in AngioQuant. For reasons of readability, significant differences are displayed in the graph if they occurred between the approaches (VEGF, Suramin or *w*/*o*) on the same material (* *p* ≤ 0.05; ** *p* ≤ 0.01; *** *p* ≤ 0.001; **** *p* ≤ 0.0001). Standard deviation is indicated by error bars.

The effect of different approaches was analyzed by comparing samples cultured on the same material and receiving different supplements. As expected, significant differences in the length and number of junctions were found between vascular endothelial growth factor (VEGF) and suramin supplementation on all three materials (Figure 4B,C). On tissue culture plate (TCPS) and gel with copolymer, length and number of branches were significantly higher in the VEGF approach than without supplements. Quantification showed that within each approach, the mean length of structures formed and the mean number of branches were higher on gels than on TCPS. The results show that the copolymer-modified fibrin hydrogel has a positive effect on angiogenesis. Comparing the TCPS against the fibrin hydrogel condition, the fibrin hydrogel triggers angiogenesis in every case. On fibrin hydrogels, a clearly strengthened network can be seen with and without the use of copolymer. With the use of copolymer-modified fibrin hydrogel, longer branches and higher interconnectivity are achieved. It is noteworthy that the addition of the inhibitor suramin on TCPS completely prevents angiogenesis, resulting in the typical islet formation (Figure 4A). On the copolymer-modified fibrin hydrogel, capillaries are formed, even in this condition, to a higher degree. This shows the positive influence of the new copolymer; if this is added to the gel, the branch length and the number of junctions is increased significantly (Figure 4B,C). In the VEGF approach, the results are similar, both with copolymer and without, as the fibrin gel as a material in combination with the growth factor generally stimulates and supports angiogenesis. Remarkably, when suramin is added to the sample as an inhibitor, the use of the copolymer in the gel continues to have a significantly positive effect on angiogenesis. Despite the inhibitor, we were able to achieve an increased number of junctions and prolonged branch length with our newly modified gel. The reason for this could be that the use of the copolymer, which is associated with mechanical controllability, which increases the stiffness of the gel, so that there is a positive influence on angiogenesis. This effect continues over the inhibition by suramin.

### 2.3. Parallel Alignment of Capillary-like Structures on Hydrogel Surfaces

**Figure 5:**
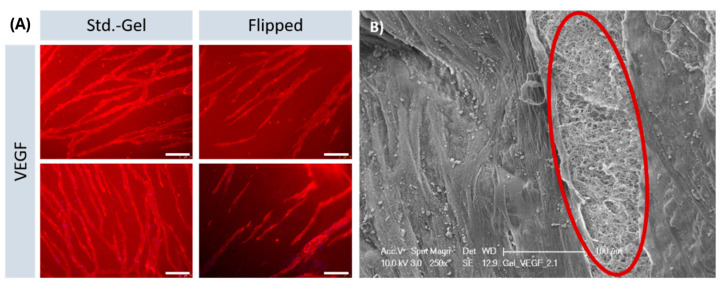
Alignment of capillaries on hydrogel surfaces. (**A**) Immunofluorescence staining against CD31 shows capillaries oriented in parallel on hydrogel surfaces, irrespective of the surface curvature (“Std.-Gel” indicates the regular procedure of gel fabrication, “Flipped” indicates the gels that were removed from the wells and inverted to eliminate the surface curvature). Images shown are from four different wells at 100x magnification. Scale bars: 150 µm. (**B**) SEM image showing parallel orientation of capillaries on the surface and amorphous arrangement of fibrin inside the gel (circled in red). White dot-like residues on the surface were identified as buffer-remnants by EDX-analysis.

The parallel alignment of the capillaries on the fibrin hydrogels was remarkable. Known from the state of the art, patterning and alignment of endothelial cells on hydrogels have been reported in cases in which this behavior was actively initiated. Strategies to induce such an alignment include exposure to cyclic mechanical strain, exposure to flow, or specific scaffold design features, including endothelial cell pre-seeding on tubular structures and parallel arrangement of microfibers [20,21,22,23,24]. Possible reasons for the spontaneous alignment here could be an alignment of HUVEC along the fibrin fibers present in the gel, likely mediated by integrin-based adhesion or an alignment caused by the surface curvature of the gels. To analyze whether the alignment is a result of flexing or traction of the gel during polymerization phase, an experiment was carried out in which the gels were removed from the well plate after casting, rotated by 180 degrees and only then populated. By inverting the gels, tension is removed. Nevertheless, the capillaries that formed on the surface of flipped gels, presented the parallel orientation that has been observed before (Figure 5A). SEM images showed the surface of the gel, as well as areas in which the gel was pulled open to reveal the arrangement of fibers. Despite amorphous surfaces, the targeted alignment of the capillaries is evident.

### 2.4. Formation of Capillary-like Structures in a 3D Environment

**Figure 6:**
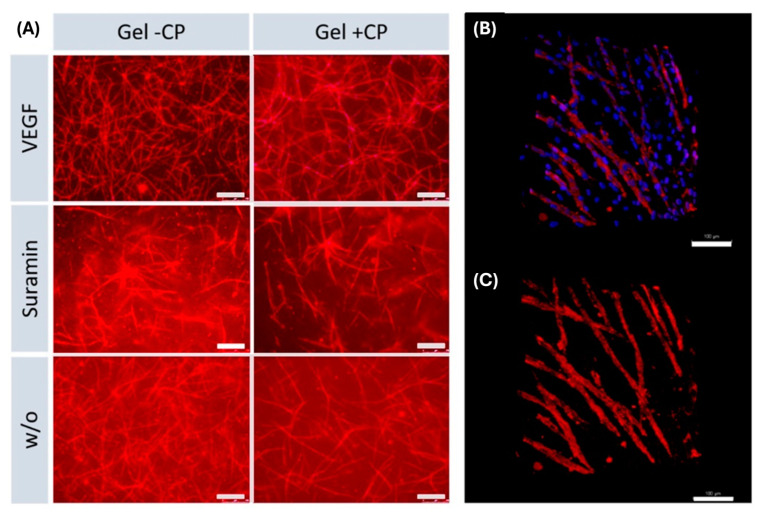
Capillary network formation inside fibrin-based hydrogels with and without copolymer. (**A**) Immunofluorescence staining of CD31 (red); images were taken at 50× magnification. Scale bars: 250 µm. (**B**,**C**) Images of a network formed in the VEGF approach in a gel with copolymer, acquired with a two-photon microscope. (**C**) Shows only the CD31 signal, whereas (**B**) also includes the DAPI signal, clearly showing that MSC are localized between capillary-like structures. Scale bars: 100 µm.

In the tissue engineering field, the transfer to three-dimensional domains is of great importance. In the two-dimensional field, we could show that we achieved significantly longer branches and more junctions by using fibrin hydrogel in combination with our new copolymer. This observation should also be verified in the 3D domain. Based on the work of Kniebs et al., the number of cells used for resuspension in the fibrinogen solution was increased for 3D approaches to 5.25 × 10^5^ cells per cell type per well [25]. With a complete translation to a 3D co-culture system, both MSC and HUVEC were suspended in the fibrinogen solution before polymerization. Successful capillary network formation was observed in all approaches (Figure 6). We were able to show that even in the multidimensional range, our fibrin hydrogel supports angiogenesis well and delivers stable results. Capillaries were formed to a lesser extent with the addition of suramin but were also present in this approach despite the inhibitor.

### 2.5. Lumen Detection

**Figure 7:**
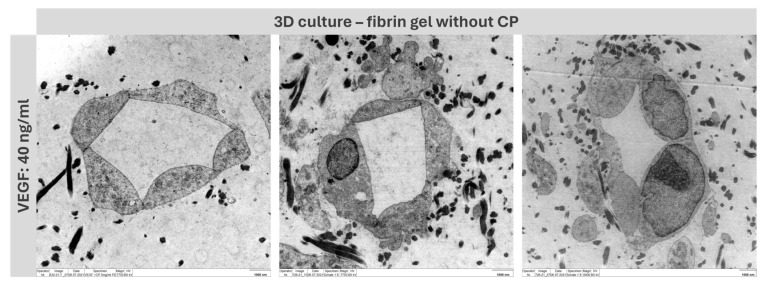
TEM analysis of the lumen of capillary-like structures in fibrin gel (5 mg/mL fibrinogen) without copolymer with an added VEGF concentration of 40 ng/mL. Scale bar: 1000 nm.

To prove that the tubes formed functional capillaries, it was essential to analyze the lumen of the structure. For this purpose, a three-dimensional co-culture of MSC and HUVEC was established in fibrin-based hydrogels without copolymer in a preliminary experiment. By adding VEGF at a concentration of 40 ng/mL, stable capillary formation was achieved, which was then subsequently analyzed using transmission electron microscopy (TEM) (Figure 7).

### 2.6. Mechanical Adjustability of Fibrin Gels

**Figure 8:**
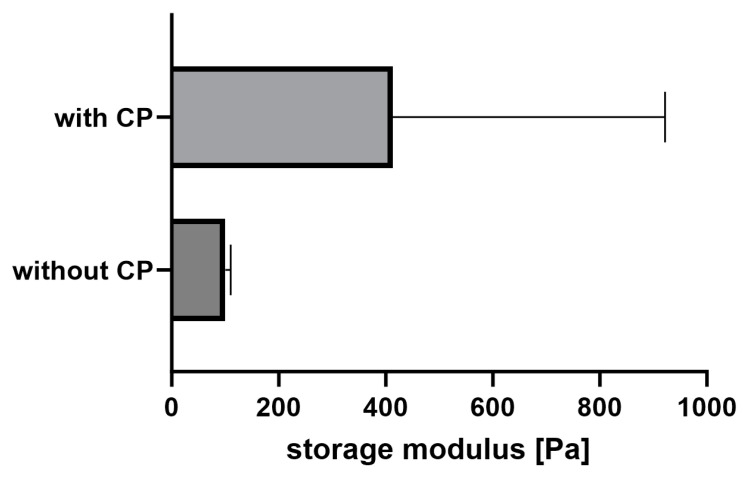
Averaged storage moduli of fibrin-based hydrogels with and without copolymer-reinforcement.

The addition of a functional copolymer does not influence the gelation properties, as shown in Figure 3A. Already at the start of the time-dependent measurement, the storage modulus is above the loss modulus, indicating that the gelation is fast and takes place before the instrument starts recording the data. The storage and the loss moduli are parallel to the *x*-axis with increasing frequency in the frequency-dependent measurements (Figure 3B). This means that both gels have viscoelastic properties. The addition of the copolymer does not influence the frequency-dependent behavior, but at a higher modulus value. Both hydrogels also show a slight strain-stiffening behavior with increasing strain (Figure 3C). This behavior is typical for biological tissues, like fibrin, to withstand forces, like the blood flow. That the reinforcement of the fibrin-based hydrogels with the functional copolymer can even enhance the strain-stiffening has already been shown in our previous work [15]. The copolymer also increases the storage modulus of the material and, therefore, its stiffness, but at a concentration of approximately 11 w%, the increase of the averaged storage modulus is not significant (Figure 8).

In our previous work, we have demonstrated that the highest storage modulus was achieved at a copolymer concentration of approximately 2 w% [15]. A further increase of the concentration was not beneficial for the storage modulus. The reason for this is that the copolymer can already react with the thiols and amines of the fibrinogen in the solution before the gelation starts. With an increasing copolymer concentration, the probability also increases that covalent bonds from the functional copolymer towards the thrombin binding site increases. In this case, the fiber formation would be disturbed, resulting in a lower storage modulus compared to those achieved at lower copolymer concentrations.

### 2.7. Capillary-like Structures on Hydrogels with and without Copolymer

**Figure 9:**
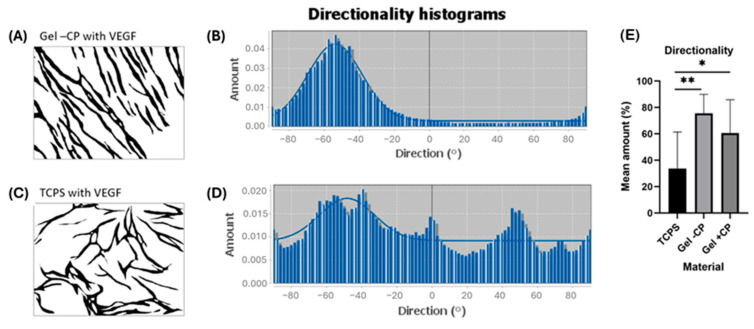
Directionality analysis with ImageJ. (**A**,**B**) Example of a pre-processed image of capillary-like structures on gel without copolymer in the VEGF approach and its corresponding histogram, showing a clear peak indicating the preferred orientation around −58° and a well-fitted curve. (**C**,**D**) Example of pre-processed image of capillary-like structures on TCPS in the VEGF approach and its corresponding histogram, which includes several spikes and a curve with a poorer fit. (**E**) Comparison of mean amounts in the VEGF approach across three materials (* *p* ≤ 0.05; ** *p* ≤ 0.01).

Remarkably, the capillary-like structures formed on the gels showed a parallel orientation, independent of donor, age, or supply of additional factors. To further characterize the alignment of capillary-like structures that was observed on both gels but not on TCPS, pre-processed pictures of the VEGF approach were used for directionality analysis with ImageJ. In short, the analysis of an image results in a histogram reflecting the number of structures in the image that are subjected to a certain angular direction. For the highest peak of the histogram, the program fits a gaussian curve and displays an “amount” value, which indicates the percentage of the present structures that align in the specific direction of the highest peak and fit under its respective curve. Two example images and histograms are given in Figure 9A–D. Capillary-like structures on TCPS and gels with and without copolymers show means of 33.7%, 60.6%, and 75.5%, respectively. Structures on both gels show a significantly higher mean, thus a significantly clearer directionality, when compared to TCPS (Figure 9E). Consequently, the parallel alignment on the fibrin gels with and without copolymer is remarkably enhanced in comparison to TCPS, confirming the impression of parallel structure orientation that occurs predominantly on gel surfaces [18]. The results show that hydrogels offer significantly improved capillarization compared to conventional cell culture plates, which is crucial for more efficient wound healing. The pronounced capillarization in hydrogels enables enhanced blood flow and nutrient delivery to the cells, thereby accelerating tissue regeneration. Sufficient capillarization is essential for ensuring rapid wound healing as it promotes tissue oxygenation, thereby supporting cellular activity and the healing process.

Comparing the formation of capillary-like structures on gels and TCPS, it was observed that the gels induced an alignment of the resulting capillaries on gels and that the gels triggered the formation of capillary-like structures to some extent, even in the suramin and *w*/*o* approaches.

The reason that capillaries arrange themselves in parallel when growing on fibrin hydrogels is likely due to the mechanical and biological properties of the hydrogel. Fibrin hydrogels have a 3D network structure that can influence the orientation and alignment of the growing capillaries. The biochemical signals in the hydrogel can direct the migration and alignment of capillary cells, leading to the formation of parallel structures [26,27]. Furthermore, factors such as oxygen gradients and nutrient availability also play a role in shaping the organization of capillaries on fibrin hydrogels.

### 2.8. Formation of Capillary-like Structures in a 3D Environment

The fact that the capillary networks formed in the hydrogels with copolymer appear less dense is because additional crosslinks provided by the copolymer improve the mechanical properties of the material and simultaneously create an ultrastructure with thicker fibrin strands that are tightly linked together, resulting in an altered porosity. A homogeneous, structured angiogenesis in a three-dimensional context is present here. In contrast to the 2D endothelial networks, images of the 3D structures could not be quantified with the previously applied method due to their multi-layered nature. Images taken with the fluorescence microscope only present a fraction of the gel’s depth and, therefore, only a fraction of the formed network. With two-photon microscopy, it is feasible to make a more detailed statement.

### 2.9. Lumen Detection

The TEM views demonstrate that the resulting structures are real capillaries with closed lumen. The individual cells arrange themselves around a lumen located in the center. However, this is, of course, only a sample location of the entire capillary; further experiments must be conducted to analyze whether the capillary has a lumen throughout and could guarantee a blood flow. Staining with Texas-red labelled dextran can be applied to allow for conclusions about the maturity of the capillaries and can reveal potential leakage [28,29].

## 3. Conclusions

In this study, in vitro angiogenesis was successfully achieved on fibrin-based hydrogels, both on the surface and within a 3D environment, resulting in the formation of capillary-like networks. Comparisons were made between network formation on tissue culture polystyrene (TCPS) and hydrogels with and without the copolymer. Co-culturing human umbilical vein endothelial cells (HUVEC) with mesenchymal stem cells (MSC) on hydrogels led to the formation of dense networks with well-oriented capillary-like structures.

The experiments also investigated the impact of incorporating the copolymer PVP_12400-co-_GMA_10mol%_ into fibrin hydrogels, showing successful formation of three-dimensional capillary-like networks. Future work may involve fabricating more complex scaffolds using 3D printing to generate functional layers and embedding specific growth factors in functionalized hydrogels for tailored applications.

Our findings demonstrate the proangiogenic properties of fibrin hydrogels and the potential for further enhancement by incorporating the copolymer. Notably, the copolymer was found to support capillary formation, even in the presence of the molecular inhibitor suramin, which could have implications for addressing poorly healing wounds [30,31].

The biohybrid system developed in this study offers adjustable mechanical properties and has the potential to address organ donor shortages and surpass conventional implants in terms of functionality, biocompatibility, and longevity. Vascularization of these implants is crucial for cell viability during in vitro maturation and for supporting growth, remodeling, and repair processes in vivo [32].

Once the interactions at the cell-biomaterial interface are fully understood, the approach of vascularizing constructs using fibrin-based hydrogels and specific copolymers may find broader applications. Exploring different copolymers and their interactions with fibrin hydrogels could open up new possibilities for biohybrid tissues in the future.

Expectations for co-culturing HUVEC and MSC on fibrin hydrogels included the formation of dense networks with well-oriented capillary-like structures, both on the surface and within the hydrogel. The incorporation of a copolymer was anticipated to enhance three-dimensional capillary-like network formation. Results confirmed these expectations, demonstrating successful in vitro angiogenesis and the formation of robust capillary networks on fibrin-based hydrogels, both on the surface and within a 3D environment. Co-culturing HUVEC and MSC led to well-oriented capillary-like structures, while the incorporation of the copolymer facilitated the formation of three-dimensional capillary-like networks. These findings validate the efficacy of the approach in promoting vascularization.

Internal studies have shown that co-culture of HUVEC and MSC promotes capillary formation and, thus, it was expected that this behavior would be enhanced by the use of a suitable scaffold material, such as fibrin gel. These expectations were confirmed and even exceeded by the present results, as the use of the copolymer further increased capillarization. It was particularly noteworthy that the use of the fibrin gel led to the molecular inhibition of suramin being overcome.

Fibrin hydrogel holds potential for tissue engineering angiogenesis but faces challenges. These include controlling degradation rates while promoting stability and optimizing mechanical properties. Uniform distribution of cells and growth factors is also crucial. Future prospects involve modifying scaffolds with copolymers and bioactive molecules, utilizing advanced fabrication techniques like 3D printing, and understanding scaffold–cellular interactions. Overcoming these challenges will maximize fibrin hydrogel’s potential in supporting angiogenesis for tissue engineering applications.

## 4. Materials and Methods

### 4.1. Preparation of Fibrin-Based Hydrogels

Fibrinogen powder (100 mg) from human plasma (62% protein, Sigma Aldrich/Merck, St. Louis, MO, USA) was diluted in 5 mL aqua ad iniectabilia and 5 mL incomplete GBSH_5_ buffer (without D-Glucose, containing 0.37 g L^−1^ KCl, 0.2 g L^−1^ MgCl_2_ in 6H_2_O, 0.15 g L^−1^ MgSO_4_ in 7H_2_O, 7.00 g L^−1^ NaCl, 0.12 g L^−1^ Na_2_HPO_4_, 1.19 g L^−1^ HEPES). Dialysis tubing was prepared by heating tubes of approximately 30 cm length in 1 mM EDTA, placed in a boiling water bath for 10 min. Tubes were rinsed with aqua ad and knotted at one end. When completely dissolved, the fibrinogen suspension was transferred to the prepared dialysis tubes; tubes were knotted at the other end, covered in incomplete GBSH_5_, and stored at 4 °C overnight. Dialysis tubes were emptied into falcon tubes and centrifuged at 5000× *g* for 30 min. The supernatant containing the fibrinogen was collected, sterile filtered, and stored in aliquots of 1 mL (6.2 mg fibrinogen/mL) at −80 °C until usage.

**Figure 10:**
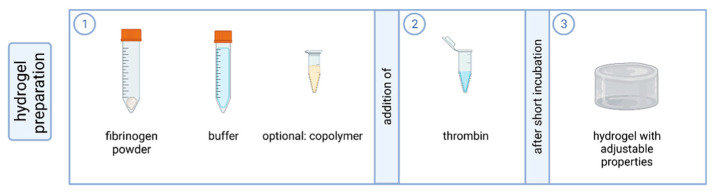
Components of the preparation of the fibrin-based hydrogels. For the preparation of a fibrin-based hydrogel with adjustable properties, the fibrinogen was mixed with buffer and optional copolymer. The addition of thrombin and uniform mixing leads to polymerization so that a solid hydrogel is produced after a short incubation period. Created with biorender.com (accessed on 23 March 2023).

A fibrinogen buffer suspension was prepared according to Table 1. For experiments with gels of both fibrin and the copolymer PVP_12400-co-_GMA_10mol%_, the copolymer was dissolved in 4.6 mL incomplete GBSH_5_ (30 mg/mL) to maintain the same overall volume of gels with and without copolymer. To prepare the gels in 24-well plates, thrombin was transferred to the bottom of the well and fibrinogen buffer suspension was added and mixed quickly [15]. Gels were incubated at 37 °C for 15 min before cell seeding (Figure 10).

### 4.2. Rheology

Rheological measurements were conducted to analyze the mechanical properties of the hydrogels. A TA Instrument Discovery HR-3 hybrid rheometer, which was equipped with a 20 mm cone plate geometry (2° cone) with a solvent trap, was used. The solutions for the hydrogel synthesis were prepared as stated above. After the addition of the thrombin solution to the mixture and a fast shaking of the vial, 74 µL of the liquid was pipetted on the Peltier plate of the rheometer, which was heated to 37 °C. The gap was adjusted to 51 µm. To avoid evaporation and drying of the hydrogel, the solvent trap was filled with some water and the setup was closed with a lid. A time-dependent measurement at a fixed frequency of 1 Hz and a fixed strain of 0.1% was performed to ensure a complete gelation of the sample. Then, at a fixed strain of 0.1%, a frequency-dependent measurement was conducted. In this experiment, the frequency was increased from 0.01 Hz to 100 Hz. Lastly, an amplitude sweep was conducted. In the strain-dependent measurement, the frequency was kept at 1 Hz and the strain was increased from 0.1 to 1000%. To analyze the reproducibility of the results, the measurements were conducted twice [15].

### 4.3. Cell Culture and Capillary Formation In Vitro

Experiments were performed with MSC and HUVEC isolated by protocols well-established in the group [33].

**Figure 11:**
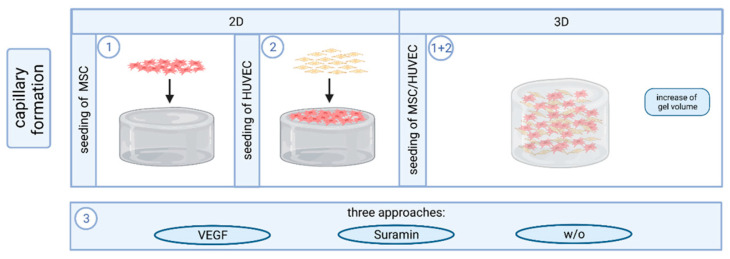
Approaches to capillary formation on and in fibrin-based hydrogels. For the analysis of angiogenesis in the two-dimensional approach, MSC were first seeded on the hydrogel surface, followed by the seeding of HUVEC on the feeder layer of the MSC. For the three-dimensional approach, the gel volume was first increased so that the HUVEC and MSC could be introduced directly into the gel as a co-culture during the polymerization phase. Subsequently, the gels were treated in three different ways, (i) by adding VEGF, (ii) by suramin, and (iii) without additives. Created with biorender.com (accessed on 23 March 2023).

Prepared hydrogels were covered with 4 × 10^4^ MSC in 400 µL SCM per well and incubated (37° C, 5% CO_2_) for two days. Then, 4 × 10^4^ HUVEC in 400 µL EGM-2 were seeded on top of the MSC layer (2D approach). Three different approaches with (i) 6 µL VEGF (2.5 µg/mL) per well, (ii) 5 µL Suramin (1 mM) per well, and (iii) untreated control were investigated in triplicate [34,35]. VEGF acts as a stimulator and suramin as an inhibitor of angiogenesis. After HUVEC seeding, cells were incubated for two hours to allow for adherence before the respective angiogenesis stimulating or inhibiting factor was added. Medium and VEGF/suramin supplementation were renewed every other day until fixation after seven days of co-culture [10]. For experiments on cells inside the gel, the cell density was adjusted to 5.25 × 10^5^ cells/mL for each cell type, gel volumes were increased to 350 µL per well; the ratio of the individual components remained unaltered (3D approach; Figure 11) [25].

### 4.4. Immunofluorescence Staining and Microscopy

Samples were fixed with 4% PFA for 60 min. Samples were washed with PBS for 45 min, blocked with 3% BSA for 60 min, and again washed with PBS for 5 min. The primary antibody (mouse anti-human anti-CD31, 1:400 in 1% BSA) was added, samples were incubated at 37 °C for 60 min and subsequently washed with PBS at 4 °C overnight. The secondary antibody (goat anti-mouse labelled with Alexa Flour 488) was applied and washed under avoidance of light exposure. Samples were treated with DAPI solution (1 µg/mL) for 5 min, then washed three times with PBS and stored at 4 °C until image acquisition at the microscope. Per cavity on a 24-well plate, 400 µL PFA, PBS, and 3% BSA were pipetted; the dilutions of the first and second antibody were used at volumes of 200 µL per well. Unless indicated differently, all steps were carried out at room temperature. The third well of each approach was used as a control for autofluorescence and unspecific binding and was, as such, only treated with the secondary antibody. When HUVEC were seeded inside the gels, the incubation with the primary and secondary antibody was extended to an overnight incubation. For qualitative analysis, three images, roughly on one line from left to right, were taken per well at 100× magnification at the fluorescence microscope. One image was taken per control well. For quantitative analysis, five images per well were taken in positions that were set in advance to sample inspection.

To assess the gel ultrastructure and lumen formation in capillary-like structures, samples were also visualized by scanning electron microscopy (SEM) and transmission electron microscopy (TEM). Visible remnants on SEM images were characterized using energy-dispersive X-ray spectroscopy (EDX).

### 4.5. VEGF Detection

During the angiogenesis assays, supernatant medium was collected every second day after the seeding of MSC. One pooled sample was collected for each approach; VEGF concentration was measured in duplicate by ELISA. Maxisorp plates (96-well) were coated with capture antibody overnight and the ELISA was carried out according to the manufacturer’s protocol (R&D Systems, Wiesbaden, Germany).

### 4.6. Quantification and Statistical Analysis

Images were processed in GIMP 2.10.12 to remove background and cell monolayers and to create an inverted binary image of the present capillary-like structures. MATLAB AngioQuant was used to analyze the total length of the network in pixels, as well as the total number of junctions. Threshold settings in AngioQuant were adjusted manually (low threshold: 130, high threshold: 200) to ensure that all structures were recognized in the skeletonized images. The same fixed thresholds were applied to all images in the batch analysis. Directionality analysis was carried out using the directionality plugin for ImageJ. GraphPad Prism 9.1.2 was used for graphs and statistical analyses. Two-way ANOVA was applied (α = 0.05) with a Tukey post hoc test.

## Figures and Tables

**Table 1:** Composition of fibrin-based hydrogels. Quantities given for 24-well plates. For gels with the copolymer, the copolymer was dissolved in 4.6 mL GBSH5 to maintain the overall volume.

Component	µL per Well
Fibrinogen (6.2 mg/mL)	207
CaCl_2_ (50 mM)	11.5
GBSH5 incomplete or PVP_12400-co-_GMA_10mol%_ in GBSH5 complete (30 mg/mL)	4.6
Tranexamic acid (100 mg/mL)	4.6
Thrombin (10U)	23

## Data Availability

The data presented in this study are available on request from the corresponding author (accurately indicate status).
